# Ascl1/Mash1 Is a Novel Target of Gli2 during Gli2-Induced Neurogenesis in P19 EC Cells

**DOI:** 10.1371/journal.pone.0019174

**Published:** 2011-04-29

**Authors:** Anastassia Voronova, Anna Fischer, Tammy Ryan, Ashraf Al Madhoun, Ilona Sylvia Skerjanc

**Affiliations:** Department of Biochemistry, Microbiology and Immunology, Faculty of Medicine, University of Ottawa, Ottawa, Canada; Center for Regenerative Therapies Dresden, Germany

## Abstract

The Sonic Hedgehog (Shh) signaling pathway is important for neurogenesis in vivo. Gli transcription factors, effector proteins of the Shh signaling pathway, have neurogenic properties in vivo, which are still poorly understood. To study the molecular basis of neurogenic properties of Gli2, we used a well-established embryonic stem cell model, the P19 embryonal carcinoma (EC) cell line, which can be induced to differentiate into neurons in the presence of retinoic acid (RA). We found that, in the absence of RA, overexpression of Gli2 induced P19 EC cells to differentiate into neurons, but not astrocytes during the first ten days of differentiation. To our knowledge, this is the first indication that the expression of Gli factors can convert EC cells into neurons. Furthermore, Gli2 upregulated expression of the neurogenic basic helix-loop-helix (bHLH) factors, such as NeuroD, Neurog1 and Ascl1/Mash1 in P19 EC cells. Using chromatin immunoprecipitation assays, we showed that Gli2 bound to multiple regulatory regions in the *Ascl1* gene, including promoter and enhancer regions during Gli2-induced neurogenesis. In addition, Gli2 activated the *Ascl1/Mash1* promoter in vitro. Using the expression of a dominant-negative form of Gli2, fused to the Engrailed repression domain, we observed a reduction in gliogenesis and a significant downregulation of the bHLH factors Ascl1/Mash1, Neurog1 and NeuroD, leading to delayed neurogenesis in P19 EC cells, further supporting the hypothesis that Ascl1/Mash1 is a direct target of Gli2. In summary, Gli2 is sufficient to induce neurogenesis in P19 stem cells at least in part by directly upregulating Ascl1/Mash1. Our results provide mechanistic insight into the neurogenic properties of Gli2 in vitro, and offer novel plausible explanations for its in vivo neurogenic properties.

## Introduction

Central nervous system (CNS) development is orchestrated by numerous signaling pathways, including the Shh signaling pathway, which in mammals is mediated by the transcription factors Gli 1, 2, and 3 (reviewed in [1]–[3]). During neurogenesis *in vivo*, Shh-mediated signaling in the notochord and floor plate is essential and sufficient for the specification of ventral cell types in CNS [4]–[9]. Based on mammalian knockout (KO) experiments reviewed in [1], Gli1 is a transcriptional activator that is dependent on Gli2 and/or Gli3-mediated transcription [1]. Gli2 is a primary mediator of Shh signaling and mainly functions as a transcriptional activator [1], however, it was shown to have repressor functions in CNS and skeletal muscle development [10], [11]. Gli3 is mainly a transcriptional repressor [1], but it also has been shown to have activator functions in embryonic development [9]–[11].

Gli proteins are known to have individual as well as combinatorial functions [12]. Although Gli1 KO mice do not exhibit any phenotype [13], zebrafish embryos lacking Gli1 show partial ventral CNS patterning defects [14]. Mice lacking Gli3 protein function exhibit neural tube closure defects [15], [16]. Dysregulation of Gli2 is lethal and causes complete loss of floor plate and reduction of V3 interneurons [17], [18]. Complimentary functions of Gli proteins are evidenced by their ability to rescue, at least in part, each other's KO phenotype [9], [11], [13], [19]. Moreover, Gli proteins were recently shown to cooperate during neurogenesis *in vivo*, creating a dynamic physical network [20]. Thus, all Gli proteins participate in early CNS development; however, teasing out the specific roles for each Gli factor has been somewhat complicated.

All Gli proteins have neurogenic properties *in vivo* as demonstrated by several studies [9], [10], [20], [21]. *Xenopus* embryos injected with Gli1, Gli2 or Gli3 showed concentration-dependent ectopic neurogenesis. Of the three family members, Gli2 had the strongest neurogenic properties [21]. It was later found that Gli2 can induce formation of motor neurons while inhibiting floorplate and neural crest differentiation [10]. In a recent study, Gli2, as well as other Gli factors, were shown to regulate the expression of some neurogenic basic helix-loop-helix (bHLH) genes such as Ncam, Neurog1 and NeuroD [20]. This correlates with the expression profile of Gli proteins in animal cap and neural plate primordium, which precedes the expression of neurogenic bHLH genes [22]. This expression pattern is also observed during neurogenesis *in vitro*, where expression of Gli transcription factors coincides with expression of Sox1/2 [23], followed by expression of NeuroD1 (referred to as NeuroD herein), Ascl1 (also known as Mash1) and culminating in NeuN and β-III tubulin (Tuj1) [24]–[26].

Ascl1 belongs to bHLH transcription factors of the *achaete-scute* family and is important for the successful differentiation of neural progenitors *in vivo* [27]–[30]. Ascl1 has recently gained new attention as a master-regulator of neurogenesis *in vitro* [31]. Ascl1 was shown to convert mouse embryonic and postnatal fibroblasts into induced neurons [31], complementing previously described induction of neurogenesis in P19 EC cells [32]. Ascl1 has also been proposed to be a downstream target of Shh signaling in adult neural progenitor cells [33], although whether the effect is direct or indirect is unknown.

Although the neurogenic properties of Gli transcription factors in primary neurogenesis have been established [10], [20], [21], the mechanistic insight into how Gli factors regulate the expression of neurogenic bHLH genes, such as Ascl1, and induction of neurogenesis, remains unknown. Since Gli2 was shown to have the strongest neurogenic properties in *Xenopus* [21], we aimed to study the molecular mechanism of Gli2-induced neurogenesis in a well-established embryonic stem cell model, the P19 EC cell line. P19 EC cells are isolated from a teratocarcinoma created by the transplantation of E7.5 mouse embryo cells into the testes of a C3H/He mouse [34]. P19 EC cells resemble mouse embryonic stem (mES) cells as they maintain a pluripotent, undifferentiated state when cultured, and can differentiate into three germ layers, ectoderm, endoderm and mesoderm upon addition of various chemical stimuli [34]–[36]. When P19 EC embryoid bodies are treated with RA, they differentiate into neurons on day 6, and astrocytes on day 10 [35]. Neurogenesis in P19 cells has been extensively studied [24], [37]–[40] and is similar to neurogenesis in mES cells [41], [42]. In this study we have found that overexpression of Gli2 induced neurogenesis, but not gliogenesis, in P19 EC cells during the first ten days of differentiation. We also found that Gli2 induced the expression of neurogenic bHLH factors such as NeuroD, Neurog1 and Ascl1. Conversely, a repressive dominant-negative Gli2 factor resulted in decreased gliogenesis and downregulated expression of NeuroD, Neurog1 and Ascl1 leading to delayed neurogenesis in P19 EC cells. Finally, Gli2 was found to bind directly to *Ascl1* gene regulatory elements during Gli2-induced neurogenesis in P19 EC cells and was able to activate the *Ascl1* promoter *in vitro*. Therefore, expression of Gli2 can convert EC cells into neurons at least in part through the direct upregulation of Ascl1.

## Materials and Methods

### P19 EC cell culture

P19 EC cells (ATCC, #CRL-1825) and P19 EC cells stably overexpressing either Gli2, a dominant negative fusion protein of Gli2 with the engrailed repression domain, or an empty vector, termed P19[Gli2], P19[Gli/EnR], or P19[Control], respectively, were described in [43]. Cells were cultured as described previously [44] and differentiated in 1% DMSO (vehicle) (Sigma-Aldrich, Canada) with or without 0.5 or 1 µM RA (Sigma Aldrich, Canada) as in [45], [46]. Briefly, cells were aggregated or cultured in monolayer in the presence of chemical stimuli at the density of 100,000 cells/ml. RA and/or DMSO was added for the first 4 days of aggregation or throughout monolayer differentiation. Media was changed every other day.

### Immunofluorescence

Antigenic analysis of differentiated cells was performed using neurofilament 68- (NF68) (Sigma-Aldrich, Canada), Tuj1- (β III tubulin) (Research Diagnostics, MA) or glial fibrillary acidic protein- (GFAP) (Zymed Laboratories, CA) specific antibodies as described in [47]–[49]. Cy3- or FITC-conjugated secondary antibodies (Jackson Immuno Research Laboratories, USA) were used for detection of indirect immunofluorescence. Briefly, cells were fixed using ice-cold methanol or 4 percent paraformaldehyde (PFA) (Fischer Scientific, Canada), and incubated with primary and secondary antibodies in phosphate buffer saline (PBS) with or without 3% BSA (Serologicals Proteins Inc, IL) and 0.3% Triton X-100 (Bio-Rad Laboratories, Canada). Hoechst dye was used as a nuclear marker. Indirect immunofluorescence was captured using a Leica DMI6000B microscope (Leica Microsystems GmbH, Germany). Images were collected at 400x magnification using a Hamamatsu Orca AG camera (Hamamatsu Photonics, Germany) and processed using Velocity 4.3.2 software (Perkin Elmer, Canada).

### Quantitative Polymerase Chain Reaction (QPCR) analysis

RNA from differentiating P19 EC cells was harvested using RNeasy Mini Kit (Qiagen, Canada) and analyzed using real-time quantitative PCR (QPCR) as described in [48], [50]. Briefly, 1 µg of RNA was reverse-transcribed (RT) to synthesize cDNA using Quantitect Reverse Transcription Kit (Qiagen, Canada). One-twentieth of the RT reaction was used as a template for QPCR amplification using the specific primers listed in Table 1 and the FastStart SYBR Green kit (Roche Applied Sciences, Canada) or Promega GoTaq qPCR Master Mix (Promega, WI). Data was acquired using ABI7300 and ABI7500 QPCR (Applied Biosystems, CA) or Eppendorf Realplex2 (Eppendorf, Canada) instruments, normalized to β-actin and analyzed as described in [51]. Data represents mean ± SEM from at least two independent biological experiments and using two clonal populations per cell line.

**Table 1:** Oligonucleotide sequences of primers utilized for real-time QPCR.

Target	Forward primer	Reverse Primer
Ascl1	ACTTGAACTCTATGGCGGGTT	CCAGTTGGTAAAGTCCAGCAG
β-actin	AAATCGTGCGTGACATCAAA	AAGGAAGGCTGGAAAAGAGC
GFAP	CCAAGCCAAACACGAAGCTAA	CATTTGCCGCTCTAGGGACTC
Gli/EnR	GGAGAGTGTGGAGGCCAGTA	CTGGGTTCCGGCTGTCTCT
Gli1	CCAAGCCAACTTTATGTCAGGG	AGCCCGCTTCTTTGTTAATTTGA
Gli2	CAACGCCTACTCTCCCAGAC	GAGCCTTGATGTACTGTACCAC
Gli3	AGCAACCAGGAGCCTGAAGTC	GTCTTGAGTAGGCTTTTGTGC
MEF2C	TCTGTCTGGCTTCAACACTG	TGGTGGTACGGTCTCTAGGA
Nanog	TCTTCCTGGTCCCCACAGTTT	GCAAGAATAGTTCTCGGGATGAA
Nestin	CCCTGAAGTCGAGGAGCTG	CTGCTGCACCTCTAAGCGA
NeuroD	GCATGCACGGGCTGAACGC	GGGATGCACCGGGAAGGAAG
Neurog1	CCAGCGACACTGAGTCCTG	CGGGCCATAGGTGAAGTCTT
Sox2	GACAGCTACGCGCACATGA	GGTGCATCGGTTGCATCTG

### Chromatin immunoprecipitation (ChIP) analysis

150 µg of chromatin from day 4 differentiating P19[Gli2] cells in the absence of RA was immunoprecipitated using 2 µg of Gli2-specific (Santa Cruz, G-20) or goat IgG non-specific antibodies (Invitrogen, Canada) and analyzed as described in [52]. Briefly, cells were cross-linked with 4 percent formaldehyde (Fischer Scientific, Canada) and chromatin was sheared as described in [52]. Sheared chromatin was incubated with Gli2 or IgG antibodies and the immune complexes were captured using protein G sepharose beads as described in [52]. Gli2 or IgG-bound chromatin was quantified as a percent chromatin input using QPCR analysis as described above. Data represents mean ± SEM from three independent biological experiments. Primers listed in Table 2 were designed for specific conserved Gli binding motifs, which were identified as described in [53].

**Table 2:** Oligonucleotide sequences of primers utilized for ChIP experiments.

Target gene	Forward primer	Reverse Primer
Ascl1 A	CTGGACTCACTGGGTGGTCT	AGAGGCTGCTAGCCATGTGT
Ascl1 B	TCTTTCTCTGTCGCCATTCA	GGACGCTCCGGTTTGTATAG
Ascl1 C	TTCTTTGAGGCCTCTTCTTCA	TGAAATGCTGACCTCTTCCA
Ascl1 D	CCTAAGATCAATGGGCCAAA	CCCACCCAACTGTCCTAGAG
Gli1	GCACCCCCTCTCTAGCTTCTATC	GGACCACCCGCGAGAAGCGCAAACT

### Immunoblot analysis

P19[Control] and P19[Gli2] cells were differentiated without RA as described above. On days 0, 4, 6 and 9 cells were washed twice with ice-cold PBS and lysed with RIPA buffer containing 1x protease inhibitor cocktail (Roche, Canada) and 0.5 mM phenylmethanesulfonylfluoride (PMSF) (Sigma-Aldrich, Canada). Lysates were clarified by centrifugation for 15 min at 13 krpm. 20 µg of total protein was resolved using 4–12% gradient NUPAGE gels (Invitrogen, Canada) according to the manufacturer's protocol using MOPS SDS running buffer. Resolved proteins were transferred to polyvinylidene fluoride (PVDF) or nitrocellulose membranes, blocked in 5% milk, and reacted with Gli2- [54], NF68- (Sigma-Aldrich, Canada), α-tubulin- (Sigma-Aldrich, Canada) or β-actin-specific antibodies (Sigma-Aldrich, Canada). Signal was detected using Horseradish Peroxidase (HRP)-conjugated secondary anti-mouse (Cell Signalling, MA) or anti-rabbit (Santa Cruz, CA) antibodies, followed by a chemiluminescence reaction using Pierce ECL substrate (Fisher Scientific, Canada).

### Ascl1 promoter analysis

HEK-293 cells were plated at a density of 300,000 cells per 35 mm tissue culture grade dish and transiently co-transfected 24 h later using FuGENE (Promega, WI) with a total amount of 4 µg of DNA with or without Gli2 and/or Gli/EnR expression plasmid described in [43] and a luciferase expression vector driven by Ascl1-8 kb promoter (termed Ascl1-luc) described in [55]. Transfection efficiency was monitored by transfecting Renilla as described in [52]. 24 h after transfection, cells were washed twice with ice-cold PBS and lysed according to the Dual Luciferase Kit protocol (Promega, WI). Luciferase activity was assayed using 10–15 µl of lysate and LmaxII384 luminometer (Molecular Devices, USA).

### Statistical analysis

ANOVA followed by post-hoc Tukey HSD test was performed using XLSTAT software (Addinsoft, NY) to determine statistical significance between mean values of two groups (*, p<0.05; **, p<0.01).

## Results

### Gli2 is expressed during neurogenesis in P19 EC cells

**Figure 1:**
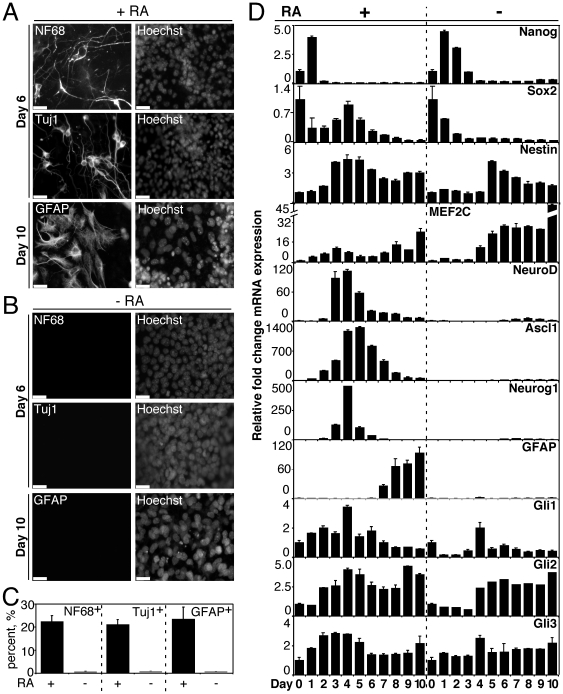
Induction of neurogenesis in P19 EC cells by RA. P19 cells were differentiated using embryoid bodies in the presence of RA as described in [35]. (**A**): Formation of Tuj1-, and NF68- positive cells with neuronal morphology on day 6 and GFAP-positive cells with astrocyte morphology on day 10 of RA-induced (+RA) differentiation. Nuclei were stained with Hoechst, scale bar is 30 µM. (**B**): P19 EC cells fail to form Tuj1-, NF68- and GFAP- positive cells in the absence of RA (-RA) on the days indicated. Nuclei were stained with Hoechst, scale bar is 30 µM. (**C**): Tuj1-, NF68- and GFAP-positive cells from (A–B) were counted in 10 random fields and expressed as % of the total number of nuclei (3,000 nuclei). (**D**): The temporal pattern of expression of indicated genes during P19 EC differentiation +/−RA. Representative QPCR analysis is shown in which fold changes are relative to day 0. Error bars represent +/− SEM.

We first sought to determine whether Gli2 is expressed during endogenous P19 EC neurogenesis. P19 EC cells were aggregated for 4 days in the presence of DMSO, with or without RA, and then plated into tissue culture dishes in the absence of drug. Cells were fixed on days 6 and 10 for examination by immunofluorescence. P19 cells were able to differentiate into Tuj1- and NF68- positive neurons by day 6 as well as GFAP-positive astrocytes by day 10 in the presence, but not in the absence, of RA (Fig. 1A–1C), in accordance with previous reports [35].

Neurogenesis was also followed by QPCR analysis of the expression of several neurogenic markers as well as by the loss of embryonic stem cell pluripotency markers, Nanog and Sox2 [56], [57] (Fig. 1D) during a 10-day time course of P19 cell differentiation with (+RA) and without RA (-RA). Expression of Nanog and Sox2 was downregulated by days 1–2 or 2–3 of differentiation + or −RA, respectively (Fig. 1D, panels Nanog and Sox2). Thus P19 EC cells lost pluripotency markers during differentiation under both conditions. Furthermore, Sox2 is also a marker of neural progenitor cells *in vitro* [58] and Sox2 transcripts were detected on days 3–5 of differentiation +RA but not –RA, supporting the RA-induction of neural progenitors cells in these cultures (Fig. 1D, panel Sox2). Expression of Nestin, which is present in neural, glial and muscle progenitor cells [59], [60], was upregulated by day 3 or 5 of differentiation + or – RA, respectively (Fig. 1D, panel Nestin). Notably, MEF2C, which is expressed during P19 EC neurogenesis [61], [62], was upregulated on days 1–3 of differentiation +RA but not –RA (Fig. 1D, Panel MEF2C). Subsequent upregulation of MEF2C on days 4–10 could be indicative of cardiac or skeletal myogenesis in –RA differentiation [63]. Expression of the neuronal bHLH factors, NeuroD, Ascl1, and Neurog1, peaked from days 3–5 of differentiation +RA but not - RA (Fig. 1D, panels NeuroD, Ascl1 and Neurog1). Therefore, neuronal markers were expressed during days 3–5 and their expression was specific to RA-induced differentiation of P19 cells.

The expression of GFAP, a glial marker [35], was specific to RA-induced differentiation and was upregulated starting at day 7 (Fig. 1D, panel GFAP). Transcription factors Gli1-3 were expressed throughout the differentiation and were elevated during days 2–6 of RA-induced differentiation (Fig. 1D, panels Gli1, Gli2 and Gli3). Therefore, Gli factors, including Gli2, are expressed during P19 EC neurogenesis. The summary of gene expression from Fig. 1 is listed in Table 3.

**Table 3:** Summary of gene expression for P19 cells treated + and − RA, P19[Gli2] cells treated - RA, and P19[Gli/EnR] cells treated + RA.

Cell line and treatment	Gli1	Gli2	Gli3	Gli/EnR	Nanog	Sox2	Nestin	MEF2C	NeuroD	Ascl1	Neurog1	GFAP	Ref
P19 + RA	+	+	+	N/A	−	+	+	+	+	+	+	+	Fig. 1D
P19 - RA	+	+	+	N/A	−	−	+	+	−	−	−	−	Fig. 1D
P19[Gli2] -RA	+	++	+	N/A	−	+/−	+	+#	+	+	+	+/−	Fig. 3 and unpublished observations
P19[Gli/En] +RA	−	−	−	++	+/−*	−	−	+	−	−	−	−	Fig. 5

“++” means high upregulation as a result of overexpression, “+” means upregulation, “+/−” means no change, and “−” means downregulation of gene expression as compared to day 0. “N/A” means not applicable. For P19[Gli2] and P19[Gli/EnR] cell lines gene expression was compared to their respective control cell lines.

*Expression of Nanog was downregulated only in undifferentiated P19[Gli/EnR] cells;

# Voronova and Skerjanc, unpublished observations.

### Gli2 upregulates expression of neurogenic bHLH factors and induces neurogenesis in P19 EC cells

To test whether Gli2 has neurogenic properties in stem cells, we first aimed to establish a stem cell model, where parental differentiating stem cells would fail to undergo neurogenesis. If overexpression of Gli2 resulted in neurogenesis in the context of this model, it would indicate that Gli2 possessed neurogenic properties *in vitro*. Based on the results from Fig. 1, -RA differentiation was chosen to study the effect of Gli2 on neurogenesis.

**Figure 2:**
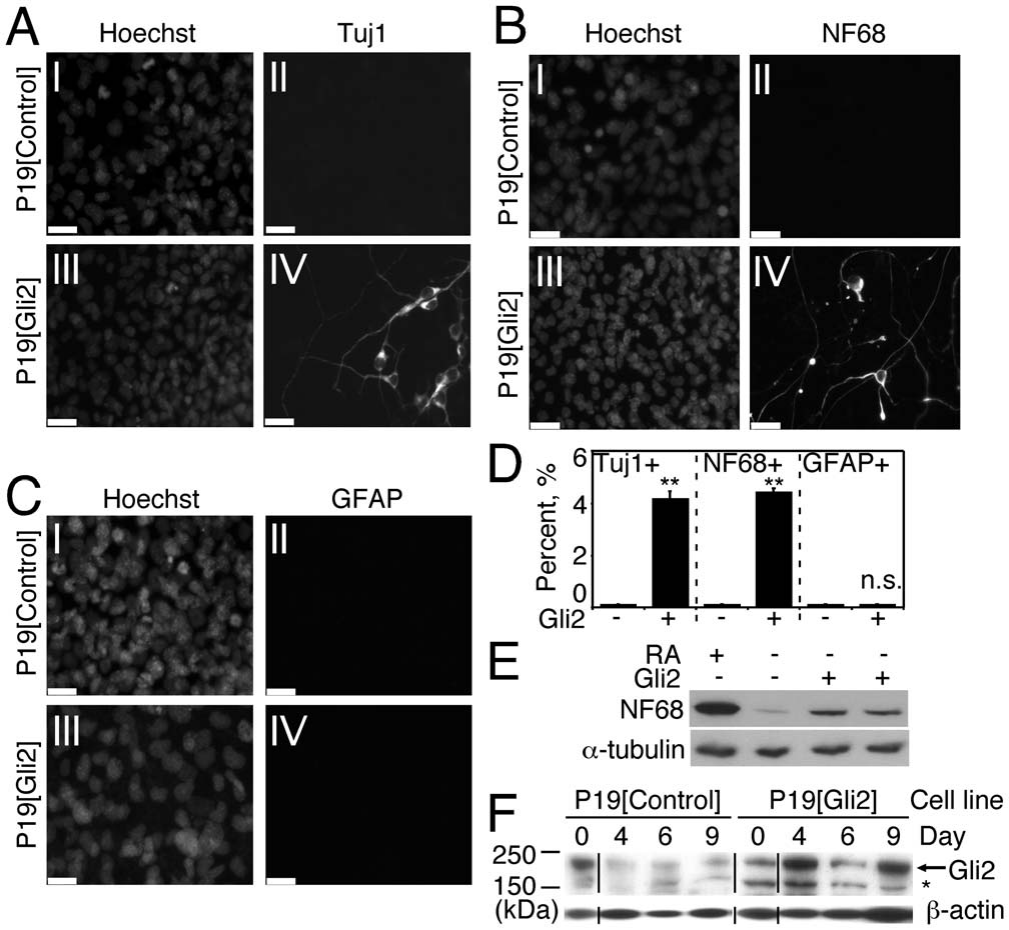
Expression of Gli2 induces neurogenesis in P19 EC cells. (A–C): P19[Gli2] and P19[Control] cells were stained with Tuj1 and NF68 antibodies on day 6 or GFAP antibodies on day 10 of –RA differentiation. Nuclei were stained with Hoechst, scale bar is 30 µM. (**D**): Tuj1-, NF68- and GFAP-positive cells from (A)–(C) were counted in 10 random fields and expressed as a percentage of the total number of nuclei (10,000 cells; n = 4) (**p<0.01, n.s.  =  not significant). (**E**): NF68 immunoblot using total protein from day 6 differentiated P19, P19[Control] and two clonal populations of P19[Gli2] cell lines. P19 cells were differentiated in the presence of RA and served as a positive control. α-tubulin served as a loading control. (**F**): Total protein from –RA differentiating P19[Control] and P19[Gli2] cells was harvested on the days indicated, separated and immunoblotted with Gli2-specific antibodies. β-actin served as a loading control. Asterisk denotes non-specific binding of Gli2 antibodies.

We stably overexpressed Gli2 in P19 EC cells, termed P19[Gli2], and examined P19[Gli2] cells differentiated –RA for the presence of neurogenic markers by immunofluorescence and western blot analysis (Fig. 2). On day 6 of differentiation, Tuj1- and NF68-positive cells with neuronal morphology were seen in P19[Gli2] cultures, indicating that neurogenesis was indeed induced (Fig. 2A, panels III and IV and Fig. 2B, panels III and IV). P19[Control] cells failed to undergo neurogenesis under the same conditions (Fig. 2A, panels I and II and Fig. 2B, panels I and II). On day 10 of differentiation, the absence of GFAP-positive cells in both P19[Control] (Fig. 2C, panels I and II) and P19[Gli2] (Fig. 2C, panels III and IV) cells indicated no or delayed gliogenesis. Thus, overexpression of Gli2 induced neurogenesis but not gliogenesis in aggregated P19 cells in the first ten days of differentiation.

To estimate the extent of neurogenesis induced by exogenous Gli2, Tuj1- and NF68-positive cells were counted and normalized to the number of Hoechst stained nuclei. P19[Gli2] cells differentiated into neurons by day 6, and they represented about 4 percent of total cells (Fig. 2D). No neurons were detected in P19[Control] cells differentiated under the same conditions (Fig. 2D). This result was confirmed by immunoblot analysis using NF68 antibodies, which showed an induction of NF68 protein in two clonal populations of P19[Gli2] cells when compared to the P19[Control] cell line (Fig. 2E). P19 EC cells differentiated in the presence of RA served as a positive control (Fig. 2E). Gli2 protein expression was confirmed by western blot analysis to be at higher levels in P19[Gli2] cells compared to control cells on days 4, 6, and 9, with the highest levels of Gli2 protein observed on day 4 (Fig. 2F). Thus, the expression of exogenous Gli2 in P19 EC cells leads to induction of neurogenesis, thereby confirming the neurogenic properties of Gli2 *in vitro*.

**Figure 3:**
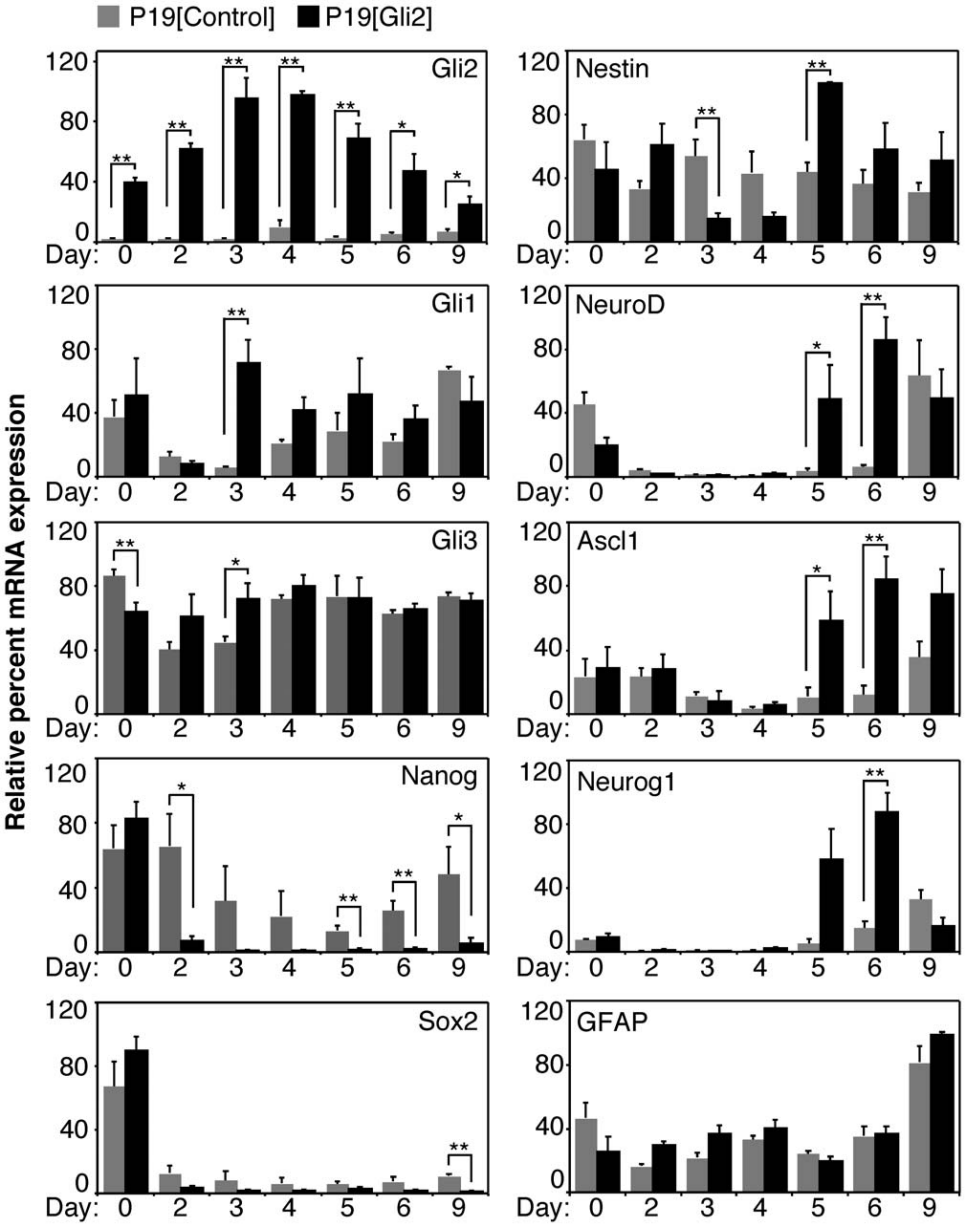
Expression of Gli2 induces expression of neuronal bHLH factors. Expression of indicated genes was assayed by QPCR analysis, n = 4. RNA from differentiating P19[Control] (grey bars) and P19[Gli2] cells (black bars) was harvested on days 0, 2–6 and 9 of differentiation without RA. Error bars represent +/− SEM from at least two biological replicas using two clonal populations (*p<0.05, **p<0.01).

To determine the expression pattern of neuronal markers induced by Gli2, we performed a time-course of QPCR gene expression analysis of markers from Fig. 1D. Overexpression of Gli2 was fairly stable throughout the differentiation (Fig. 3, panel Gli2). Upregulation of Gli1 and Gli3 expression on day 3 in P19[Gli2] cells as compared to the control cell line (Fig. 3, panels Gli1 and Gli3) suggested that overexpression of Gli2 activated the Shh signaling pathway. The expression of Nanog was significantly decreased in P19[Gli2] cells by day 2 (Fig. 3, panel Nanog), compared to P19[Control] cells, resembling the accelerated loss of Nanog observed during RA-induced differentiation (Fig. 1D). Sox2 was downregulated in both P19[Control] and P19[Gli2] cells by day 2 (Fig. 3, panel Sox2), indicating a loss of pluripotency.

Notably, later expression of Sox2 in P19[Gli2] and control cells remained low during most of the differentiation, suggesting that Gli2 did not induce neurogenesis via Sox2 upregulation (Fig. 3, panel Sox2). Furthermore, the expression of Nestin, which is expressed in neuro-glial and muscle progenitor cells [59], [60], was significantly downregulated on day 3, but upregulated on day 5 in P19[Gli2] cells (Fig. 3, panel Nestin). Finally, the expression of neurogenic bHLH factors NeuroD, Ascl1 and Neurog1 was upregulated by overexpression of Gli2 by days 5 or 6 (Fig. 3, panels NeuroD, Ascl1 and Neurog1). This correlated with the induction of neurogenesis as observed in Fig. 2. The expression of GFAP was not changed by overexpression of Gli2 even on day 9 of differentiation (Fig. 3, panel GFAP), which correlated with the absence of GFAP-positive cells on day 10 of differentiation (Fig. 2C). Gene expression analysis is summarized in Table 3. Thus, overexpression of Gli2 induced the expression of neuronal markers by days 5 and 6 of differentiation while gliogenesis was unaffected in the first ten days of differentiation.

### Expression of dominant-negative Gli/EnR delays neurogenesis, reduces gliogenesis and reduces expression of neurogenic bHLH factors in P19 EC cells

**Figure 4:**
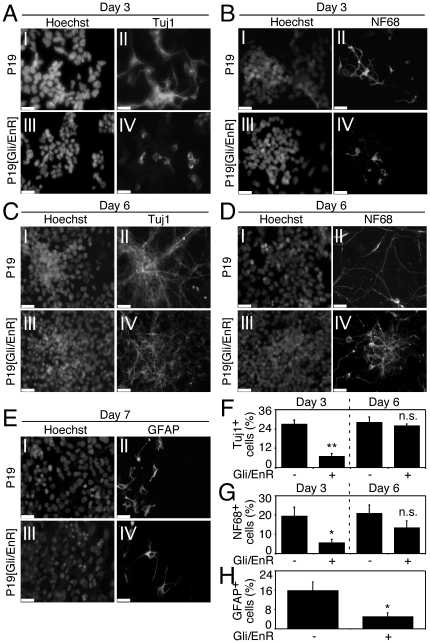
Expression of Gli/EnR delays neurogenesis and decreases gliogenesis in P19 EC cells. Cells were differentiated using a monolayer procedure described in [46] in the presence of RA. (**A–D**): Day 3 or day 6 differentiated P19[Gli/EnR] and P19 cells were stained with Tuj1- or NF68-specific antibodies. (**E**): Day 7 differentiated P19[Gli/EnR] and P19 cells were stained with GFAP-specific antibodies. Nuclei were stained with Hoechst, scale bar is 30 µM. (**F–H**): Tuj1-, NF68- and GFAP-positive cells from (A-E) were counted in 10 random fields and normalized with the number of nuclei (10,000 cells; n = 4), *p<0.05, **p<0.01, n.s.  =  not significant.

Since Gli factors play complimentary roles [12], we utilized a Gli2 dominant-negative construct, created by fusing the Gli2 DNA binding domain to the Engrailed repressor domain, termed Gli/EnR. Gli/EnR would bind to the Gli DNA binding domain and recruit repressors, inhibiting transcription in a fashion that cannot be rescued by other Gli factors, such as Gli1 or Gli3 [64], [65]. Parental P19 and P19[Gli/EnR] cells were differentiated in the presence of RA using a monolayer procedure as described in [46], where the formation of neurons is detected within 3 days, and the formation of astrocytes is detected within 7 days. Antigenic analysis revealed a decrease in Tuj1- and NF68-positive neurons as compared to control P19 cells on day 3 of differentiation (Fig. 4A, 4B, 4F and 4G). However, by day 6, the levels of Tuj1- and NF68-positive neurons were similar (Fig. 4C, 4D, 4F and 4G), indicating that expression of Gli/EnR in P19 EC cells resulted in delayed neurogenesis. Day 7 differentiated P19[Gli/EnR] cultures showed a decrease in GFAP-positive astrocytes as compared to P19 control cells (Fig. 4E and 4H).

**Figure 5:**
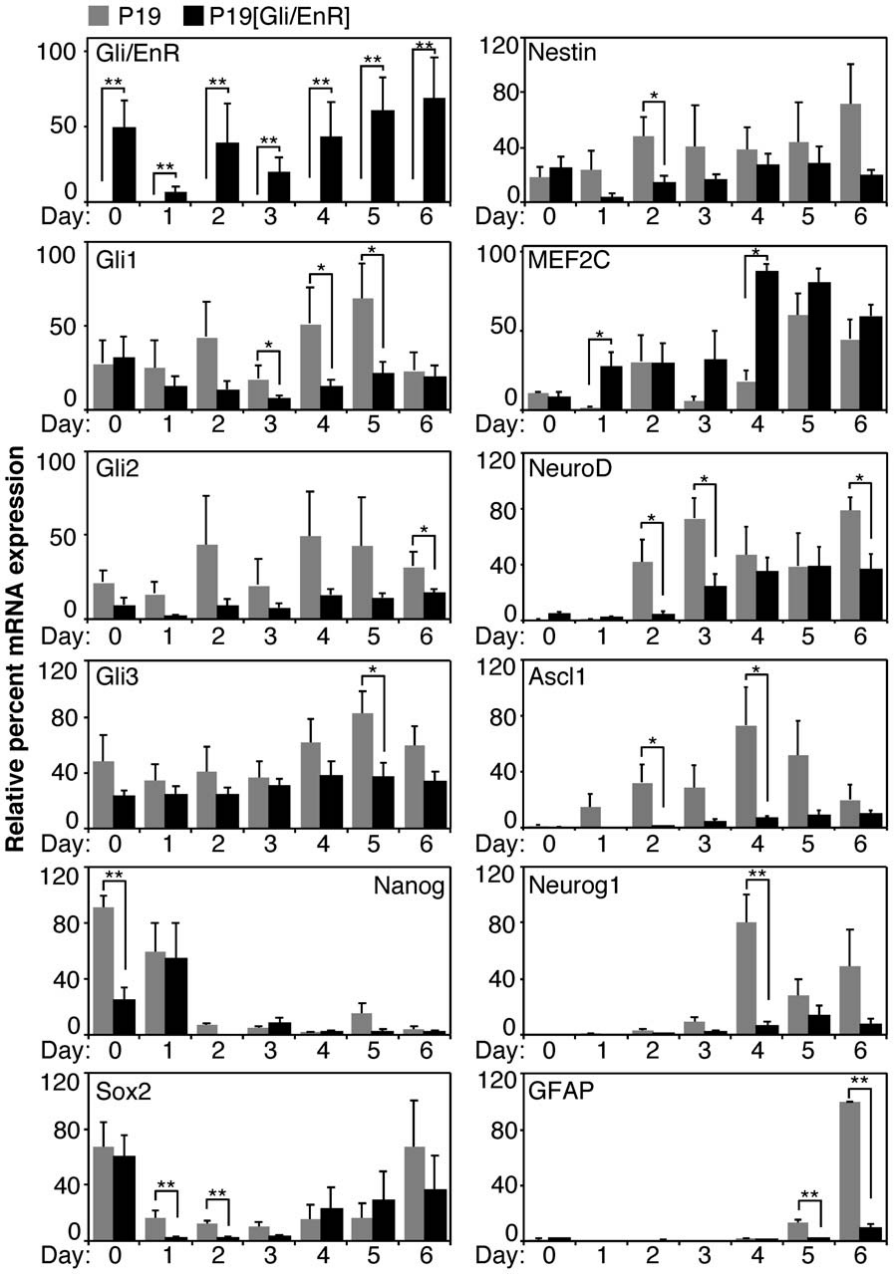
Expression of Gli/EnR reduces expression of neuronal bHLH factors. Expression of indicated genes was assayed by QPCR analysis (Gli1, Gli3, n = 8; Gli2, MEF2C, n = 6; Gli/EnR, Sox2, Nestin, Ascl1, Neurog1, GFAP, n = 4; NeuroD, n = 3) by QPCR analysis. RNA from differentiating P19 (grey bars) and P19[Gli/EnR] cells (black bars) was harvested on days 0–6 +RA differentiation. Error bars represent +/− SEM from at least three biological replicas using two clonal populations (*p<0.05, **p<0.01).

To determine the expression pattern of neuronal markers affected by expression of Gli/EnR, we performed a time-course of QPCR gene expression analysis using markers from Fig. 1D. Gli/EnR was fairly stably overexpressed throughout the differentiation (Fig. 5, panel Gli/EnR). Downregulation of Gli1, Gli2 and Gli3 in P19[Gli/EnR] cells as compared to P19 control cells confirmed suppression of the Shh signaling pathway (Fig. 5, panels Gli1, Gli2 and Gli3).

The expression of Nanog, a direct target of Gli2 in neural stem cells [66], was significantly downregulated by the expression of Gli/EnR in undifferentiated cells (Fig. 5, panel Nanog, day 0), but was relatively unchanged by Gli/EnR expression during differentiation (Fig. 5, panel Nanog, days 1–6). Another direct target of Gli2 in neural stem cells, Sox2 [67], was expressed at the same level in undifferentiated P19[Gli/EnR] and P19 control cells (Fig. 5, panel Sox2, day 0). However, Sox2 was significantly downregulated by the expression of Gli/EnR on days 1 and 2 of differentiation (Fig. 5, panel Sox2). There was a trend in downregulation of the expression of Nestin in P19[Gli/EnR] cultures throughout the differentiation, however, the decrease in the Nestin mRNA levels was only statistically significant (p<0.05) on day 2 of differentiation (Fig. 5, panel Nestin). Thus, the neural progenitor markers Sox2 and Nestin were downregulated predominantly on days 1 and 2 of differentiation by dominant-negative Gli2 expression.

Surprisingly, expression of MEF2C was upregulated on days 1 and 4 in P19[Gli/EnR] cells as compared to P19 control cells (Fig. 5, panel MEF2C). Since MEF2C can initiate neurogenesis and upregulate Ascl1 expression [47], it is possible that MEF2C may compensate, at least partially, for the Gli/EnR inhibition of neurogenesis.

The neurogenic bHLH factors, NeuroD, Ascl1, and Neurog1 were downregulated by the expression of Gli/EnR throughout the timecourse and were most significantly downregulated ranging from days 2–4 (Fig. 5, panels NeuroD, Ascl1, and Neurog1). By day 6 the extent of downregulation lessened with only NeuroD remaining significantly downregulated, suggesting a delay in neurogenesis rather than an inhibition, in agreement with the immunofluorescence analysis.

**Figure 6:**
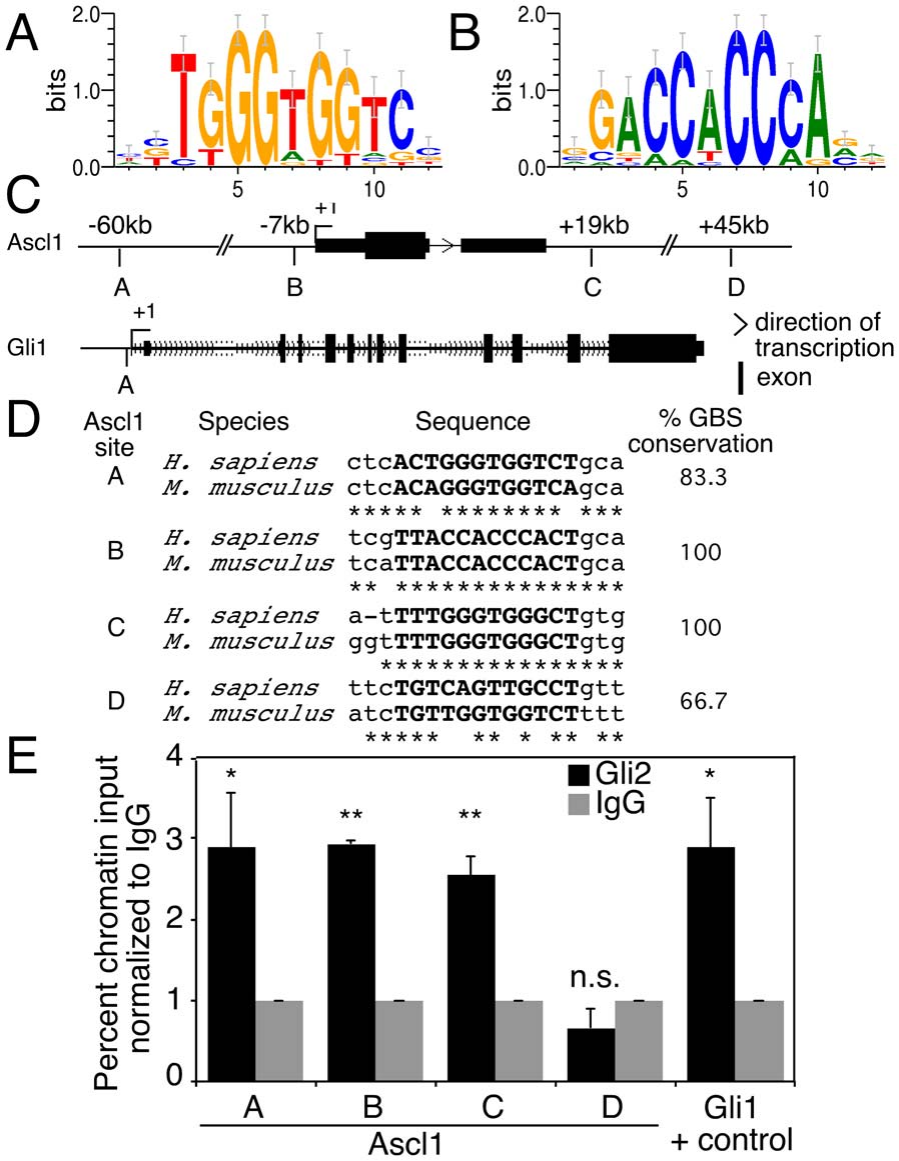
Gli2 binds Ascl1 gene regulatory elements in P19 EC cells. (A–B): TRANSFAC (#M01037) Gli binding motif in forward and reverse direction, respectively. (**C**): Custom tracks of Ascl1 and Gli1 genes using UCSC genome browser (http://genome.ucsc.edu). Triangles designate the direction of transcription, and black boxes designate exons. The Ascl1 gene (+/− 100 kb) from mouse and human genomes was searched for conserved theoretical Gli binding as described in [53], which are designated as A–D. Their positions relative to the transcriptional start site (+1) are indicated as numbers. The known Gli binding site in the Gli1 gene is designated as A [68] (**D**): Comparison of mouse and human sequences of Ascl1 A–D sites from (C). The sequence of the Gli binding site (GBS) is marked in bold. (**E**): ChIP analysis showing enrichment by Gli2 antibodies of Ascl1 chromatin fragments corresponding to sites A–C, from (C). Sheared chromatin from day 4 -RA differentiated P19[Gli2] cells was immunopurified using Gli2-specific (black bars) or IgG non-specific (grey bars) antibodies. The Gli1 promoter served as a positive control. Percent chromatin input was calculated using QPCR analysis and primers listed in Table 2. Error bars represent +/− SEM from three biological replicas (*p<0.05, **p<0.01, n.s.  =  not significant).

The expression of GFAP was severely downregulated in P19[Gli/EnR] cells on days 5 and 6 of differentiation (Fig. 5, panel GFAP), which correlated with a decrease in GFAP-positive cells in P19[Gli/EnR] cultures (Fig. 6H). Thus both the immunofluorescence and the gene expression analysis support an inhibition of gliogenesis by dominant negative Gli2 expression. The summary of gene expression from Fig. 5 is listed in Table 3.

In summary, a dominant negative Gli2 mutant attenuated neurogenesis in P19 EC cells shown by the downregulation of the neurogenic bHLH factors. In addition gliogenesis was inhibited, as shown by the downregulation of GFAP.

### Gli2 binds to Ascl1 gene regulatory elements and activates its promoter

Since overexpression of Gli2 elevated the expression of several neurogenic bHLH genes, including Ascl1, (Fig. 3, panel Ascl1), which was previously proposed to be a downstream target of the Shh signaling pathway [33], we were interested whether Gli2 could bind directly to the Ascl1 gene regulatory elements. *In silico* analysis of the *Ascl1* gene using the TRANSFAC Gli binding motif (Fig. 6A and 6B) revealed 4 theoretical, conserved Gli binding sites both upstream and downstream of the transcriptional start site (Fig. 6C and 6D), suggesting that *Ascl1* might be a novel direct target of Gli2. Since day 4 differentiating P19[Gli2] cells showed the highest expression of Gli2 mRNA (Fig. 3, panel Gli2) and protein (Fig. 2F), this time point was chosen for ChIP analysis using Gli2-specific antibodies or IgG-nonspecific antibodies. We observed an enrichment of chromatin fragments corresponding to the *Ascl1* A–C sites, but not to the *Ascl1* D site (Fig. 6E) with Gli2 antibodies, as compared to non-specific IgG antibodies. The *Gli1* promoter was used as a positive control based on a previous report [68] (Fig. 6E). Thus, Gli2 binds directly to multiple sites located up- and downstream of the *Ascl1* gene.

**Figure 7:**
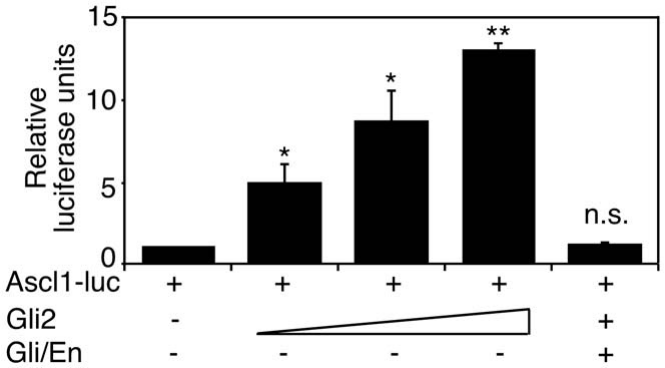
Gli2 activates the Ascl1 promoter. HEK-293 cells were transiently cotransfected with or without Gli2 and a construct containing the Ascl1 8 kb promoter driving the luciferase gene (Ascl1-luc) in ratios 2∶1, 4∶1 and 6∶1 relative to Ascl1-luc. Equal parts of Gli/EnR were transfected together with Gli2 at a ratio of 4∶1 relative to Ascl1-luc. Fold changes are relative to Ascl1-luc activity with the Ascl1-luc plasmid alone. Error bars represent +/− SEM from three biological replicas (*p<0.05, **p<0.01). No significant (n.s.) increase was observed in the presence of Gli/EnR.

To assess the functionality of the ChIP results, we performed *Ascl1* promoter analysis with Gli2. The *Ascl1* B site is located within the *Ascl1* promoter region, which has been characterized previously and contains 3 additional, non-conserved Gli binding sites [55]. Promoter studies revealed that Gli2 directly activated the *Ascl1* promoter in a concentration-dependent manner up to 13 (±1) fold (Fig. 7). Gli/EnR suppressed activation of the *Ascl1* promoter by Gli2, confirming the ability of Gli/EnR to bind to the Gli binding sequences and act as a repressor (Fig. 7). Thus, Gli2 elevates expression of Ascl1, binds directly to its gene regulatory regions and activates its promoter *in vitro*.

## Discussion

In this paper we have shown, for the first time, that overexpression of Gli2 induced neurogenesis, but not gliogenesis, in P19 EC cells during the first ten days of differentiation. We have also shown that Gli2 regulated the expression of neurogenic bHLH factors like NeuroD, Neurog1 and Ascl1 during Gli2-induced neurogenesis in P19 EC cells. The expression of repressive Gli/EnR resulted in a delay of P19 EC neurogenesis, as well as decrease in gliogenesis. The expression of neurogenic bHLH factors including Ascl1 was also decreased by the expression of Gli/EnR. Additionally, Gli2 directly bound to *Ascl1* gene regulatory elements during P19 EC Gli2-induced neurogenesis, and activated the *Ascl1* promoter *in vitro*. To our knowledge, this is the first indication that Gli factors can directly regulate neurogenic bHLH factor expression.

Our finding that Gli2 could induce neurogenesis supports and extends previous studies [9], [10], [20], [21]. This is the first indication that expression of Gli2 induces, rather than enhances neurogenesis in an embryonic stem cell model. For example, other publications have demonstrated a 3–10 fold enhancement of neurogenesis by application of Shh agonist to mES [41] or by overexpression of Gli1 in hES cells [69]. In our study, we observed an induction of neurogenesis from 0% of neurons in the control cell line to 4% in P19[Gli2] cells. Furthermore, the extent of induction of neurogenesis caused by expression of Gli2 in our study is similar to the extent of neurogenesis caused by other transcription factors [32], [47] and to that seen in mES and hES cells [41], [69]. In contrast, mES and hES cells spontaneously differentiate into neurons [70], [71] and thus can only be used to study the enhancement of neurogenesis but not the induction by exogenous stimuli.

Our finding that expression of a dominant-negative Gli2 in P19 EC cells results in delayed neurogenesis supports and extends a previous study, where inhibition of the Shh signaling pathway in human ES cells resulted in reduced formation of Tuj1-positive neurons [72]. Since the authors only tested one time-point for the presence of Tuj1-positive neurons, it is possible that a later time-point would reveal a restored amount of neurons in cultures treated with cyclopamine [72].

The expression patterns of Gli1-3 during P19 EC neurogenesis *in vitro* shown in this study is supported by previous work showing a role for Shh signaling in mES cell neurogenesis [23]. Relatively low fold changes of upregulation for Gli2 (4 fold), as compared to Ascl1 (1400 fold) or NeuroD (120 fold), are due to high levels of Gli2 expression in undifferentiated cells (Fig. 2F) [73]. Since Gli factors are expressed in multiple lineages, including myogenesis [43], [74] and neurogenesis [23], their expression is not specific to RA-induced neurogenesis (Fig. 1D). This is similar to expression of Nestin, which is present in both muscle and neuronal precursor cells [59], [60]. Furthermore, a major effect of Shh signaling is the activation of Gli2 function, as opposed to the upregulation of Gli2 expression [75], [76].

It was previously shown that Gli proteins upregulated the expression of neurogenic bHLH factors such as Ncam, Neurog1 and NeuroD in *Xenopus* [20], however, the molecular mechanism for this phenomenon was not elucidated. Using P19[Gli2] cells, we were able to confirm the ability of Gli2 to elevate the expression of neurogenic bHLH factors (Fig. 3), although with a slight delay as compared to RA-induced expression of these factors (Fig. 1D). Moreover, the expression of dominant-negative Gli2 resulted in significant downregulation of NeuroD, Neurog1 and Ascl1 expression. This result correlates with a previous report showing reduced expression of Ascl1 in neural progenitor cells treated with cyclopamine [33]. In this study, Gli2 directly bound to the conserved Gli sites A–C in the *Ascl1* gene. The *Ascl1* promoter, which contains the B site, was activated by Gli2 in a concentration-dependent manner. The *Ascl1* C site falls within a novel highly conserved enhancer that directs expression in the eye (http://enhancer.lbl.gov) [77]. The Gli proteins along with the Shh ligand are important in eye development in *Xenopus* [78], [79], mouse [5], [80], [81] and human [82]. *Ascl1* D site was the least conserved between mouse and human (Fig. 6B), and did not appear to be bound by Gli2. Therefore, Gli2 upregulates Ascl1 expression, binds to its gene regulatory elements and activates its promoter during Gli2-induced neurogenesis in P19 EC cells.

Notably, the *Ascl1* gene was not reported to be bound by Gli1 in a genome-wide ChIP-microarray analysis of mES cells undergoing neurogenesis [83], however its expression was reported to be attenuated by Shh in both mES [83] and adult neural stem cells [33]. Furthermore, it is possible that *Ascl1* gene element(s) were bound by Gli1 in differentiating mES cells, but were not included in the results due to high false-discovery rate cutoff in reported ChIP-microarray analysis [83]. Finally, it is possible that *Ascl1* is a direct target of Gli2, and not Gli1, during neurogenesis *in vitro*.

While Nanog is a direct target of Gli2 in adult neural stem cells [66] we did not observe easily explained changes in Nanog expression in differentiating P19[Gli2] or P19[Gli/EnR] cells. Under pluripotent monolayer conditions, Gli/EnR inhibited Nanog expression (Fig. 5), but Gli2 did not change Nanog expression (Fig. 3). The latter phenomenon might be explained by the similar levels of Gli2 protein expression in pluripotent undifferentiated P19[Control] and P19[Gli2] cells (Fig. 2F, day 0). In contrast, during differentiation, Gli2 enhanced Nanog downregulation, whereas Gli/EnR did not affect Nanog expression. These results are likely due to the difficulty of comparing results in postnatal rats [66] to an embryonic stem cell model [34], which is heterogeneous and encompasses different developmental stages, including pluripotent stem cells, neural progenitors, and neurons. Thus, while Gli2 function is important for maintaining stem cell Nanog expression, it cannot further enhance it. Pluripotency was maintained, despite the decrease in Nanog expression, in part because Sox2 was still expressed (Fig. 5). Further, P19[Gli/EnR] cells could still differentiate into cardiac muscle (Voronova and Skerjanc, unpublished observations) and neurons (Fig. 4).

Sox2 was also shown to be a direct target of Gli2 during differentiation of neural stem cells derived from E14.5 murine telencephalon [67]. Although gain- or loss-of-function of Gli2 did not affect Sox2 expression in the pluripotent monolayer stem cell stage, loss of Gli2 function delayed Sox2 upregulation at the neural progenitor stage (Fig. 5). Notably, expression of Gli2 did not upregulate Sox2 or Nestin mRNA at the predicted progenitor stage, although Nestin was upregulated later, at the same time as the bHLH neurogenic genes (Fig. 3). It is possible that Gli2 upregulated the expression of other Sox factors, like Sox1 and Sox3, which exhibit redundant biological functions [84]. These results suggest that Gli2 may bypass the progenitor stage and induce neurogenesis through upregulation of the bHLH neurogenic genes.

Surprisingly, the levels of MEF2C were upregulated in P19 cells overexpressing Gli/EnR. Since MEF2C was shown to initiate neurogenesis and drive Ascl1 expression [47], as well as have anti-apoptotic functions important for the survival of cells during neurogenesis [62], [85], [86], it is possible that MEF2C is able to compensate for the Gli/EnR inhibition of neurogenesis. Notably, on day 4 when MEF2C is greatly upregulated by Gli/EnR, Ascl1 is downregulated (Fig. 5), suggesting that MEF2C cannot bypass the inhibition of Ascl1 by dominant negative Gli2 at this time point. Since Gli/EnR is an active dominant negative mutant, it is possible that MEF2C could compensate for the simple loss of Gli2 signaling if Gli2 was knocked-down or -out. The relatively mild phenotype of P19[Gli/EnR] cells is consistent with previous reports showing that Shh signaling is not essential for the neural tube development [1], [17], [18].

The overexpression of Gli2 did not result in the formation of astrocytes in the first ten days of differentiation, whereas P19[Gli/EnR] cells showed reduced gliogenesis. Previous reports have demonstrated increased astrocyte formation in hES cultures differentiated in the presence of cyclopamine [72]. The discrepancy in results might be due to the dominant-negative repressive effect of Gli/EnR, which is capable of overriding the activity of Gli factors (Fig. 7). Cyclopamine, on the other hand, binds Smo, and thus prevents activation of Gli transcription factors by Shh [87]. However, other signaling molecules have been implicated in the activation of Gli factors, such as TGFβ [88] and Wnt [89]. Moreover, Zic factors have also been implicated in modulating the transcriptional activity of Gli factors as well as in binding Gli binding sites in the chromatin [90]. It is possible that expression of Gli/EnR caused a delay in gliogenesis, similar to neurogenesis, however, this hypothesis was not tested. To our knowledge, this is the first indication that dominant-negative Gli/EnR causes a delay in neurogenesis and a decrease in gliogenesis in P19 stem cells.

In summary, our findings indicate that Gli2 has neurogenic properties *in vitro*. Gli2 is able to directly regulate expression of the neurogenic bHLH factor, Ascl1, and convert P19 EC cells into neurons, but not astrocytes in the first ten days of differentiation. Dominant-negative Gli2 is able to suppress expression of neurogenic bHLH factors and delay neurogenesis. Gli2 is probably not a sole regulator of Ascl1 expression during neurogenesis, as there are several other proteins, including Notch1 [91], MEF2C [92] and Hes1 [55], which were shown to regulate Ascl1 expression. Our findings unravel new molecular mechanistic insight into the neurogenic properties of Gli2 *in vitro*, thus offering novel plausible explanations for Gli2 neurogenic properties *in vivo*.

## References

[1] Riobo NA, Manning DR (2007). Pathways of signal transduction employed by vertebrate Hedgehogs.. Biochem J.

[2] Ruiz IAA, Palma V, Dahmane N (2002). Hedgehog-Gli signalling and the growth of the brain.. Nat Rev Neurosci.

[3] Marti E, Bovolenta P (2002). Sonic hedgehog in CNS development: one signal, multiple outputs.. Trends Neurosci.

[4] Marti E, Bumcrot DA, Takada R, McMahon AP (1995). Requirement of 19K form of Sonic hedgehog for induction of distinct ventral cell types in CNS explants.. Nature.

[5] Chiang C, Litingtung Y, Lee E, Young KE, Corden JL (1996). Cyclopia and defective axial patterning in mice lacking Sonic hedgehog gene function.. Nature.

[6] Roelink H, Porter JA, Chiang C, Tanabe Y, Chang DT (1995). Floor plate and motor neuron induction by different concentrations of the amino-terminal cleavage product of sonic hedgehog autoproteolysis.. Cell.

[7] Ericson J, Morton S, Kawakami A, Roelink H, Jessell TM (1996). Two critical periods of Sonic Hedgehog signaling required for the specification of motor neuron identity.. Cell.

[8] Wijgerde M, McMahon JA, Rule M, McMahon AP (2002). A direct requirement for Hedgehog signaling for normal specification of all ventral progenitor domains in the presumptive mammalian spinal cord.. Genes Dev.

[9] Bai CB, Stephen D, Joyner AL (2004). All mouse ventral spinal cord patterning by hedgehog is Gli dependent and involves an activator function of Gli3.. Dev Cell.

[10] Ruiz i Altaba A (1998). Combinatorial Gli gene function in floor plate and neuronal inductions by Sonic hedgehog.. Development.

[11] McDermott A, Gustafsson M, Elsam T, Hui CC, Emerson CP (2005). Gli2 and Gli3 have redundant and context-dependent function in skeletal muscle formation.. Development.

[12] Lipinski RJ, Gipp JJ, Zhang J, Doles JD, Bushman W (2006). Unique and complimentary activities of the Gli transcription factors in Hedgehog signaling.. Exp Cell Res.

[13] Park HL, Bai C, Platt KA, Matise MP, Beeghly A (2000). Mouse Gli1 mutants are viable but have defects in SHH signaling in combination with a Gli2 mutation.. Development.

[14] Karlstrom RO, Tyurina OV, Kawakami A, Nishioka N, Talbot WS (2003). Genetic analysis of zebrafish gli1 and gli2 reveals divergent requirements for gli genes in vertebrate development.. Development.

[15] Theil T, Alvarez-Bolado G, Walter A, Ruther U (1999). Gli3 is required for Emx gene expression during dorsal telencephalon development.. Development.

[16] Tole S, Ragsdale CW, Grove EA (2000). Dorsoventral patterning of the telencephalon is disrupted in the mouse mutant extra-toes(J).. Dev Biol.

[17] Ding Q, Motoyama J, Gasca S, Mo R, Sasaki H (1998). Diminished Sonic hedgehog signaling and lack of floor plate differentiation in Gli2 mutant mice.. Development.

[18] Matise MP, Epstein DJ, Park HL, Platt KA, Joyner AL (1998). Gli2 is required for induction of floor plate and adjacent cells, but not most ventral neurons in the mouse central nervous system.. Development.

[19] Mo R, Freer AM, Zinyk DL, Crackower MA, Michaud J (1997). Specific and redundant functions of Gli2 and Gli3 zinc finger genes in skeletal patterning and development.. Development.

[20] Nguyen V, Chokas AL, Stecca B, Ruiz i Altaba A (2005). Cooperative requirement of the Gli proteins in neurogenesis.. Development.

[21] Brewster R, Lee J, Ruiz i Altaba A (1998). Gli/Zic factors pattern the neural plate by defining domains of cell differentiation.. Nature.

[22] Lee J, Platt KA, Censullo P, Ruiz i Altaba A (1997). Gli1 is a target of Sonic hedgehog that induces ventral neural tube development.. Development.

[23] Cai C, Thorne J, Grabel L (2008). Hedgehog serves as a mitogen and survival factor during embryonic stem cell neurogenesis.. Stem Cells.

[24] Itoh F, Nakane T, Chiba S (1997). Gene expression of MASH-1, MATH-1, neuroD and NSCL-2, basic helix-loop-helix proteins, during neural differentiation in P19 embryonal carcinoma cells.. Tohoku J Exp Med.

[25] Wakabayashi N, Kageyama R, Habu T, Doi T, Morita T (2000). A novel cis-acting element regulates HES-1 gene expression in P19 embryonal carcinoma cells treated with retinoic acid.. J Biochem.

[26] Endo M, Antonyak MA, Cerione RA (2009). Cdc42-mTOR signaling pathway controls Hes5 and Pax6 expression in retinoic acid-dependent neural differentiation.. J Biol Chem.

[27] Guillemot F, Lo LC, Johnson JE, Auerbach A, Anderson DJ (1993). Mammalian achaete-scute homolog 1 is required for the early development of olfactory and autonomic neurons.. Cell.

[28] Fode C, Ma Q, Casarosa S, Ang SL, Anderson DJ (2000). A role for neural determination genes in specifying the dorsoventral identity of telencephalic neurons.. Genes Dev.

[29] Nakada Y, Hunsaker TL, Henke RM, Johnson JE (2004). Distinct domains within Mash1 and Math1 are required for function in neuronal differentiation versus neuronal cell-type specification.. Development.

[30] Pattyn A, Guillemot F, Brunet JF (2006). Delays in neuronal differentiation in Mash1/Ascl1 mutants.. Dev Biol.

[31] Vierbuchen T, Ostermeier A, Pang ZP, Kokubu Y, Sudhof TC (2010). Direct conversion of fibroblasts to functional neurons by defined factors.. Nature.

[32] Farah MH, Olson JM, Sucic HB, Hume RI, Tapscott SJ (2000). Generation of neurons by transient expression of neural bHLH proteins in mammalian cells.. Development.

[33] Wang L, Zhang ZG, Gregg SR, Zhang RL, Jiao Z (2007). The Sonic hedgehog pathway mediates carbamylated erythropoietin-enhanced proliferation and differentiation of adult neural progenitor cells.. J Biol Chem.

[34] McBurney MW, Rogers BJ (1982). Isolation of male embryonal carcinoma cells and their chromosome replication patterns.. Dev Biol.

[35] Jones-Villeneuve EM, McBurney MW, Rogers KA, Kalnins VI (1982). Retinoic acid induces embryonal carcinoma cells to differentiate into neurons and glial cells.. J Cell Biol.

[36] McBurney MW, Jones-Villeneuve EM, Edwards MK, Anderson PJ (1982). Control of muscle and neuronal differentiation in a cultured embryonal carcinoma cell line.. Nature.

[37] Lowe B, Avila HA, Bloom FR, Gleeson M, Kusser W (2003). Quantitation of gene expression in neural precursors by reverse-transcription polymerase chain reaction using self-quenched, fluorogenic primers.. Anal Biochem.

[38] Teramoto S, Kihara-Negishi F, Sakurai T, Yamada T, Hashimoto-Tamaoki T (2005). Classification of neural differentiation-associated genes in P19 embryonal carcinoma cells by their expression patterns induced after cell aggregation and/or retinoic acid treatment.. Oncol Rep.

[39] Jin Z, Liu L, Bian W, Chen Y, Xu G (2009). Different transcription factors regulate nestin gene expression during P19 cell neural differentiation and central nervous system development.. J Biol Chem.

[40] Ulrich H, Majumder P (2006). Neurotransmitter receptor expression and activity during neuronal differentiation of embryonal carcinoma and stem cells: from basic research towards clinical applications.. Cell Prolif.

[41] Wichterle H, Lieberam I, Porter JA, Jessell TM (2002). Directed differentiation of embryonic stem cells into motor neurons.. Cell.

[42] Lang KJ, Rathjen J, Vassilieva S, Rathjen PD (2004). Differentiation of embryonic stem cells to a neural fate: a route to re-building the nervous system?. J Neurosci Res.

[43] Petropoulos H, Gianakopoulos PJ, Ridgeway AG, Skerjanc IS (2004). Disruption of Meox or Gli activity ablates skeletal myogenesis in P19 cells.. J Biol Chem.

[44] Rudnicki MA, McBurney MW (1987). Cell culture methods and induction of differentiation of embryonal carcinoma cell lines.. Teratocarcinomas and embryonic stem cells A practical approach.

[45] Ridgeway AG, Petropoulos H, Wilton S, Skerjanc IS (2000). Wnt signaling regulates the function of MyoD and myogenin.. J Biol Chem.

[46] Slack RS, Skerjanc IS, Lach B, Craig J, Jardine K (1995). Cells differentiating into neuroectoderm undergo apoptosis in the absence of functional retinoblastoma family proteins.. J Cell Biol.

[47] Skerjanc IS, Wilton S (2000). Myocyte enhancer factor 2C upregulates MASH-1 expression and induces neurogenesis in P19 cells.. FEBS Lett.

[48] Kennedy KA, Porter T, Mehta V, Ryan SD, Price F (2009). Retinoic acid enhances skeletal muscle progenitor formation and bypasses inhibition by bone morphogenetic protein 4 but not dominant negative beta-catenin.. BMC Biol.

[49] Imbeault S, Gauvin LG, Toeg HD, Pettit A, Sorbara CD (2009). The extracellular matrix controls gap junction protein expression and function in postnatal hippocampal neural progenitor cells.. BMC Neurosci.

[50] Savage J, Conley AJ, Blais A, Skerjanc IS (2009). SOX15 and SOX7 Differentially Regulate the Myogenic Program in P19 Cells.. Stem Cells.

[51] Livak KJ, Schmittgen TD (2001). Analysis of relative gene expression data using real-time quantitative PCR and the 2(-Delta Delta C(T)) Method.. Methods.

[52] Savage J, Voronova A, Mehta V, Sendi-Mukasa F, Skerjanc IS (2010). Canonical Wnt signaling regulates Foxc1/2 expression in P19 cells.. Differentiation.

[53] Ovcharenko I, Loots GG, Giardine BM, Hou M, Ma J (2005). Mulan: multiple-sequence local alignment and visualization for studying function and evolution.. Genome Res.

[54] Hu MC, Mo R, Bhella S, Wilson CW, Chuang PT (2006). GLI3-dependent transcriptional repression of Gli1, Gli2 and kidney patterning genes disrupts renal morphogenesis.. Development.

[55] Chen H, Thiagalingam A, Chopra H, Borges MW, Feder JN (1997). Conservation of the Drosophila lateral inhibition pathway in human lung cancer: a hairy-related protein (HES-1) directly represses achaete-scute homolog-1 expression.. Proc Natl Acad Sci U S A.

[56] Chambers I, Colby D, Robertson M, Nichols J, Lee S (2003). Functional expression cloning of Nanog, a pluripotency sustaining factor in embryonic stem cells.. Cell.

[57] Avilion AA, Nicolis SK, Pevny LH, Perez L, Vivian N (2003). Multipotent cell lineages in early mouse development depend on SOX2 function.. Genes Dev.

[58] Smukler SR, Runciman SB, Xu S, van der Kooy D (2006). Embryonic stem cells assume a primitive neural stem cell fate in the absence of extrinsic influences.. J Cell Biol.

[59] Wiese C, Rolletschek A, Kania G, Blyszczuk P, Tarasov KV (2004). Nestin expression–a property of multi-lineage progenitor cells?. Cell Mol Life Sci.

[60] Hockfield S, McKay RD (1985). Identification of major cell classes in the developing mammalian nervous system.. J Neurosci.

[61] Black BL, Ligon KL, Zhang Y, Olson EN (1996). Cooperative Transcriptional Activation By the Neurogenic Basic Helix-Loop-Helix Protein Mash1 and Members Of the Myocyte Enhancer Factor-2 (Mef2) Family.. J Biol Chem.

[62] Okamoto S, Krainc D, Sherman K, Lipton SA (2000). Antiapoptotic role of the p38 mitogen-activated protein kinase-myocyte enhancer factor 2 transcription factor pathway during neuronal differentiation.. Proc Natl Acad Sci U S A.

[63] Skerjanc IS (1999). Cardiac and skeletal muscle development in P19 embryonal carcinoma cells.. Trends Cardiovasc Med.

[64] Gianakopoulos PJ, Mehta V, Voronova A, Cao Y, Yao Z (2011). MyoD directly upregulates premyogenic mesoderm factors during induction of skeletal myogenesis in stem cells..

[65] Ridgeway AG, Skerjanc IS (2001). Pax3 is essential for skeletal myogenesis and the expression of Six1 and Eya2.. J Biol Chem.

[66] Po A, Ferretti E, Miele E, De Smaele E, Paganelli A (2010). Hedgehog controls neural stem cells through p53-independent regulation of Nanog.. EMBO J.

[67] Takanaga H, Tsuchida-Straeten N, Nishide K, Watanabe A, Aburatani H (2009). Gli2 is a novel regulator of sox2 expression in telencephalic neuroepithelial cells.. Stem Cells.

[68] Ikram MS, Neill GW, Regl G, Eichberger T, Frischauf AM (2004). GLI2 is expressed in normal human epidermis and BCC and induces GLI1 expression by binding to its promoter.. J Invest Dermatol.

[69] Denham M, Thompson LH, Leung J, Pebay A, Bjorklund A (2010). Gli1 is an Inducing Factor in Generating Floor Plate Progenitor Cells From Human Embryonic Stem Cells.. Stem Cells.

[70] Williams RL, Hilton DJ, Pease S, Willson TA, Stewart CL (1988). Myeloid leukaemia inhibitory factor maintains the developmental potential of embryonic stem cells.. Nature.

[71] Thomson JA, Itskovitz-Eldor J, Shapiro SS, Waknitz MA, Swiergiel JJ (1998). Embryonic stem cell lines derived from human blastocysts.. Science.

[72] Lee DS, Yu K, Rho JY, Lee E, Han JS (2006). Cyclopamine treatment of human embryonic stem cells followed by culture in human astrocyte medium promotes differentiation into nestin- and GFAP-expressing astrocytic lineage.. Life Sci.

[73] Rho JY, Yu K, Han JS, Chae JI, Koo DB (2006). Transcriptional profiling of the developmentally important signalling pathways in human embryonic stem cells.. Hum Reprod.

[74] Gianakopoulos PJ, Skerjanc IS (2005). Hedgehog signaling induces cardiomyogenesis in P19 cells.. J Biol Chem.

[75] Ohlmeyer JT, Kalderon D (1998). Hedgehog stimulates maturation of Cubitus interruptus into a labile transcriptional activator.. Nature.

[76] Tukachinsky H, Lopez LV, Salic A (2010). A mechanism for vertebrate Hedgehog signaling: recruitment to cilia and dissociation of SuFu-Gli protein complexes.. J Cell Biol.

[77] Visel A, Minovitsky S, Dubchak I, Pennacchio LA (2007). VISTA Enhancer Browser–a database of tissue-specific human enhancers.. Nucleic Acids Res.

[78] Sasagawa S, Takabatake T, Takabatake Y, Muramatsu T, Takeshima K (2002). Axes establishment during eye morphogenesis in Xenopus by coordinate and antagonistic actions of BMP4, Shh, and RA.. Genesis.

[79] Perron M, Boy S, Amato MA, Viczian A, Koebernick K (2003). A novel function for Hedgehog signalling in retinal pigment epithelium differentiation.. Development.

[80] Furimsky M, Wallace VA (2006). Complementary Gli activity mediates early patterning of the mouse visual system.. Dev Dyn.

[81] Wall DS, Mears AJ, McNeill B, Mazerolle C, Thurig S (2009). Progenitor cell proliferation in the retina is dependent on Notch-independent Sonic hedgehog/Hes1 activity.. J Cell Biol.

[82] Roessler E, Du YZ, Mullor JL, Casas E, Allen WP (2003). Loss-of-function mutations in the human GLI2 gene are associated with pituitary anomalies and holoprosencephaly-like features.. Proc Natl Acad Sci U S A.

[83] Vokes SA, Ji H, McCuine S, Tenzen T, Giles S (2007). Genomic characterization of Gli-activator targets in sonic hedgehog-mediated neural patterning.. Development.

[84] Miyagi S, Kato H, Okuda A (2009). Role of SoxB1 transcription factors in development.. Cell Mol Life Sci.

[85] Mao Z, Bonni A, Xia F, Nadal-Vicens M, Greenberg ME (1999). Neuronal activity-dependent cell survival mediated by transcription factor MEF2.. Science.

[86] Li Z, McKercher SR, Cui J, Nie Z, Soussou W (2008). Myocyte enhancer factor 2C as a neurogenic and antiapoptotic transcription factor in murine embryonic stem cells.. J Neurosci.

[87] Chen JK, Taipale J, Cooper MK, Beachy PA (2002). Inhibition of Hedgehog signaling by direct binding of cyclopamine to Smoothened.. Genes Dev.

[88] Dennler S, Andre J, Alexaki I, Li A, Magnaldo T (2007). Induction of sonic hedgehog mediators by transforming growth factor-beta: Smad3-dependent activation of Gli2 and Gli1 expression in vitro and in vivo.. Cancer Res.

[89] Borycki A, Brown AM, Emerson CP (2000). Shh and Wnt signaling pathways converge to control Gli gene activation in avian somites.. Development.

[90] Mizugishi K, Aruga J, Nakata K, Mikoshiba K (2001). Molecular properties of Zic proteins as transcriptional regulators and their relationship to GLI proteins.. J Biol Chem.

[91] Kunnimalaiyaan M, Vaccaro AM, Ndiaye MA, Chen H (2006). Overexpression of the NOTCH1 intracellular domain inhibits cell proliferation and alters the neuroendocrine phenotype of medullary thyroid cancer cells.. J Biol Chem.

[92] Elmi M, Faigle R, Yang W, Matsumoto Y, Rosenqvist E (2007). Mechanism of MASH1 induction by ASK1 and ATRA in adult neural progenitors.. Mol Cell Neurosci.

